# Efficacy and immunogenicity of a Vi-tetanus toxoid conjugate vaccine in the prevention of typhoid fever using a controlled human infection model of *Salmonella* Typhi: a randomised controlled, phase 2b trial

**DOI:** 10.1016/S0140-6736(17)32149-9

**Published:** 2017-12-02

**Authors:** Celina Jin, Malick M Gibani, Maria Moore, Helene B Juel, Elizabeth Jones, James Meiring, Victoria Harris, Jonathan Gardner, Anna Nebykova, Simon A Kerridge, Jennifer Hill, Helena Thomaides-Brears, Christoph J Blohmke, Ly-Mee Yu, Brian Angus, Andrew J Pollard

**Affiliations:** a Oxford Vaccine Group, Department of Paediatrics, University of Oxford and the NIHR Oxford Biomedical Research Centre, Oxford, UK; b Nuffield Department of Primary Care Health Sciences, University of Oxford, UK; c Nuffield Department of Clinical Medicine, University of Oxford, Oxford, UK

## Abstract

**Background:**

*Salmonella enterica* serovar Typhi (*S* Typhi) is responsible for an estimated 20 million infections and 200 000 deaths each year in resource poor regions of the world. Capsular Vi-polysaccharide-protein conjugate vaccines (Vi-conjugate vaccines) are immunogenic and can be used from infancy but there are no efficacy data for the leading candidate vaccine being considered for widespread use. To address this knowledge gap, we assessed the efficacy of a Vi-tetanus toxoid conjugate vaccine using an established human infection model of *S* Typhi.

**Methods:**

In this single-centre, randomised controlled, phase 2b study, using an established outpatient-based human typhoid infection model, we recruited healthy adult volunteers aged between 18 and 60 years, with no previous history of typhoid vaccination, infection, or prolonged residency in a typhoid-endemic region. Participants were randomly assigned (1:1:1) to receive a single dose of Vi-conjugate (Vi-TT), Vi-polysaccharide (Vi-PS), or control meningococcal vaccine with a computer-generated randomisation schedule (block size 6). Investigators and participants were masked to treatment allocation, and an unmasked team of nurses administered the vaccines. Following oral ingestion of *S* Typhi, participants were assessed with daily blood culture over a 2-week period and diagnosed with typhoid infection when meeting pre-defined criteria. The primary endpoint was the proportion of participants diagnosed with typhoid infection (ie, attack rate), defined as persistent fever of 38°C or higher for 12 h or longer or *S* Typhi bacteraemia, following oral challenge administered 1 month after Vi-vaccination (Vi-TT or Vi-PS) compared with control vaccination. Analysis was per protocol. This trial is registered with ClinicalTrials.gov, number NCT02324751, and is ongoing.

**Findings:**

Between Aug 18, 2015, and Nov 4, 2016, 112 participants were enrolled and randomly assigned; 34 to the control group, 37 to the Vi-PS group, and 41 to the Vi-TT group. 103 participants completed challenge (31 in the control group, 35 in the Vi-PS group, and 37 in the Vi-TT group) and were included in the per-protocol population. The composite criteria for typhoid diagnosis was met in 24 (77%) of 31 participants in the control group, 13 (35%) of 37 participants in the Vi-TT group, and 13 (35%) of 35 participants in the Vi-PS group to give vaccine efficacies of 54·6% (95% CI 26·8–71·8) for Vi-TT and 52·0% (23·2–70·0) for Vi-PS. Seroconversion was 100% in Vi-TT and 88·6% in Vi-PS participants, with significantly higher geometric mean titres detected 1-month post-vaccination in Vi-TT vaccinees. Four serious adverse events were reported during the conduct of the study, none of which were related to vaccination (one in the Vi-TT group and three in the Vi-PS group).

**Interpretation:**

Vi-TT is a highly immunogenic vaccine that significantly reduces typhoid fever cases when assessed using a stringent controlled model of typhoid infection. Vi-TT use has the potential to reduce both the burden of typhoid fever and associated health inequality.

**Funding:**

The Bill & Melinda Gates Foundation and the European Commission FP7 grant, Advanced Immunization Technologies (ADITEC).

## Introduction

*Salmonella enterica* subspecies *enterica* serovar Typhi (*S* Typhi) is the leading cause of enteric fever affecting 12·5–20·6 million people in regions of the world with inadequate water quality and poor sanitation,^1^, ^2^ particularly in south Asia and sub-Saharan Africa. Children are especially susceptible to infection and have a high burden of illness.^3^ Mortality is estimated at 1% and about 3% of individuals become chronic carriers.^4^, ^5^ The large burden of febrile illness associated with typhoid fever in some affected populations—eg, 15% of children with fever attending a health-care facility in Nepal during one rainy season,^6^ drives widespread over-the-counter, and prescription antibiotic use.^7^ Antimicrobial resistance (AMR) is increasingly recognised among *S* Typhi lineages spreading from south Asia to Africa, with resistance to first-line antibiotics (co-trimoxazole, ampicillin, and chloramphenicol) and fluoroquinolones, and of concern, the identification of extended-spectrum β-lactamase-producing strains, contributing to treatment failure.^8^, ^9^

**Research in context**

**Evidence before this study**

We used the 2014 Cochrane review on typhoid vaccines in addition to doing a PubMed search for clinical trials using the search terms “Vi conjugate”, “typhoid” and “vaccine”, and “efficacy”, with no language restrictions up to May 28, 2017. The first capsular Vi-polysaccharide-protein conjugate (Vi-conjugate) vaccine efficacy trial, published in 2001, estimated efficacy at 89% following two doses of a prototype Vi-rEPA vaccine (Vi conjugated to recombinant *Pseudomonas aeruginosa* exotoxin A) in children aged between 2–5 years. Since then, only one other efficacy study assessing Vi-conjugate vaccines has been published. There are no efficacy trials of the Vi-tetanus toxoid conjugate vaccine (Vi-TT), which is currently being considered by WHO for licensure. The cost and logistical difficulties associated with undertaking large field efficacy trials has contributed to the paucity of published data and has hindered the advancement of Vi-conjugate vaccines.

**Added value of this study**

This is the first study to assess the efficacy of a Vi-conjugate vaccine using a controlled human infection model of typhoid fever. We have shown that this Vi-TT vaccine is safe, highly immunogenic, and prevents 55% of typhoid infections (defined as fever ≥38°C for ≥12 h or *Salmonella enterica* serovar Typhi [*S* Typhi] bacteraemia) and up to 87% of infections, when using alternative definitions of typhoid fever (fever ≥38°C followed by *S* Typhi bacteraemia). Efficacy data from this trial will help to fill a long existing knowledge gap regarding Vi-conjugate vaccines.

**Implications of all the available evidence**

Key decision makers at WHO are due to form an opinion on the use of Vi-conjugate vaccines in October, 2017. Findings from our study support the use of Vi-conjugate vaccines as they are efficacious and safe and will assist with controlling typhoid fever in high-burden settings.

Although control of typhoid through improved water quality and adequate sanitation has been shown in North America and Europe over the past century,^10^ the infrastructure required to prevent transmission in affected regions is unlikely to be realised in the short to medium term. Immunisation could be used in population-based control programmes to reduce the disease burden and its contribution to AMR. However, currently licensed typhoid vaccines are either not immunogenic in early childhood (parenteral Vi capsular polysaccharide vaccine) or are unsuitable for administration in children younger than 5 years—eg, the oral live attenuated typhoid vaccine, Ty21a, is unsuitable for use in children younger than 5 years because of its formulation in capsules, which are difficult for young children to swallow.^11^ By contrast, typhoid conjugate vaccines (TCVs), which combine the Vi-polysaccharide capsule with a protein carrier, have improved immunological properties and can be used from early infancy.^12^, ^13^, ^14^ This has been shown with a prototype TCV, Vi-rEPA (Vi conjugated to recombinant *Pseudomonas aeruginosa* exotoxin A), which has an estimated efficacy of 89% from one field study of 2–5-year-olds receiving two doses of vaccine administered 6 weeks apart.^15^ Differences in vaccine composition, including the degree of Vi de-*O*-acetylation, have been shown to affect the immunogenicity of different TCVs.^16^ In view of this, large-scale trials to formally assess the efficacy of new TCVs, including Typbar-TCV the only vaccine undergoing WHO pre-qualification are being planned; however, results from these studies will not be reported for several years. Despite the pending results of these trials, the control of typhoid fever with TCVs will be considered by the WHO's Strategic Advisory Group of Experts in October, 2017, and subsequent decisions on financing will be made by the Global Alliance for Vaccines and Immunisation.

Controlled human infection models, in which healthy volunteers are vaccinated and then deliberately exposed to the pathogen of interest, have been used to support the development of various vaccines,^17^ such as the licensed cholera vaccine,^18^ and can be rapidly deployed, albeit in a controlled artificial setting, to assess vaccine efficacy. Here, we present the first data for the efficacy of a TCV using a controlled human infection model of typhoid fever.

## Methods

### Study design and participants

In this observer and participant-masked, randomised, controlled, phase 2b study, done at the Centre for Clinical Vaccinology and Tropical Medicine (Churchill Hospital, Oxford, UK), we recruited healthy adult volunteers aged between 18 and 60 years, with no previous history of typhoid vaccination, infection, or prolonged residency in a typhoid-endemic region. All volunteers underwent extensive medical screening, which included blood screening with baseline anti-Vi IgG assessment, and gallbladder ultrasound (see appendix pp 8–10 for full inclusion and exclusion criteria).

Written informed consent was obtained from all volunteers before enrolment. The study protocol was approved by the sponsor (University of Oxford), the South Central Oxford A Ethics Committee (14/SC/1427), and the Medicines and Healthcare Products Regulatory Agency (Eudract 2014-002978-36). The study was done in accordance with the principles of the Declaration of Helsinki and the International Council for Harmonisation Good Clinical Practice guidelines.

### Randomisation and masking

At the time of enrolment, participants were randomly assigned (1:1:1) to receive a single parenteral dose of Vi-tetanus toxoid conjugate (Vi-TT; Typbar-TCV, Bharat Biotech, Hyderabad, India), Vi-polysaccharide (Vi-PS; TYPHIM Vi, Sanofi Pasteur, Lyon, France), or control meningococcal ACWY-CRM conjugate vaccine (control; MENVEO, GlaxoSmithKline, Sovicille, Italy). Both Vi-vaccines contained 25 μg of Vi-polysaccharide per 0·5 mL dose. The randomisation schedule was generated by an independent statistician using a fixed block size of six and stratified according to baseline anti-Vi IgG titre to ensure participants with pre-existing detectable antibodies were equally distributed between vaccine groups. The allocation sequence was implemented using a randomisation system to ensure allocation concealment.

The allocation ratio was subsequently altered to account for the expiry of the investigational vaccine (Vi-TT) in June, 2016. A sensitivity analysis was done to assess the effect of bias due to changing the randomisation ratio, specifically by analysing the primary endpoint separately within those participants that were randomised under the original allocation ratio.

Investigators and participants were masked to treatment allocation, and an unmasked team of nurses administered the vaccines. Vaccine allocation and administration were coordinated by an unmasked team who were not involved in subsequent study procedures. Vaccines were prepared in a separate room and administered to participants with the concealment of the vaccine syringe to maintain participant blinding. Laboratory and clinical study teams remained masked to vaccine allocation until study unblinding.

### Procedures

Following vaccination, participants completed an online diary for 7 days to monitor local and systemic symptoms, and were assessed in clinic at days 1, 3, 7, and 10.

About 1 month post-vaccination, participants were challenged orally with 1–5 × 10^4^ colony forming units (CFUs) of *S* Typhi Quailes strain (a wild-type strain originally isolated from a chronic carrier in Baltimore, MD, USA). Immediately before challenge administration, participants ingested 120 mL of sodium bicarbonate solution to neutralise gastric acid.^19^ Following challenge, participants were seen daily for vital sign measurement, blood collection, and general assessment in an outpatient clinic for a 2 week period, and completed an online diary with twice daily self-reported temperature measurements for 21 days, covering the 2 week challenge period and an additional 7 days after challenge to monitor antibiotic tolerability and symptom resolution.

Typhoid fever was diagnosed if pre-determined criteria were met: a positive blood culture with *S* Typhi collected more than 72 h post-challenge or a fever of 38°C or higher persisting for 12 h or longer (participants were denied access to antipyretics before diagnosis). Diagnosed participants commenced a 2 week course of antibiotics, either ciprofloxacin 500 mg twice daily or azithromycin 500 mg daily, and attended five follow-up appointments to monitor disease resolution. Participants diagnosed on the basis of positive blood culture were commenced on treatment when blood cultures flagged positive with Gram-negative bacilli (with confirmatory identification of *S* Typhi done after treatment commencement). Participants who did not develop typhoid fever despite oral challenge (undiagnosed) were treated with antibiotics at the end of the challenge period (day 14).

### Outcomes

The primary endpoint was the proportion of participants diagnosed with typhoid infection (ie, attack rate), defined as persistent fever of 38°C or higher for 12 h or longer or *S* Typhi bacteraemia, following oral challenge administered 1 month after Vi-vaccination (Vi-TT or Vi-PS) compared with control vaccination.

Secondary safety outcomes included vaccine tolerability and typhoid disease severity based on severity scoring of solicited events within 7 days post-vaccination and 21 days post-challenge.

Additional secondary outcomes included time to typhoid diagnosis, time to first fever, time to bacteraemia, and microbiological outcomes, such as quantitative blood culture and stool culture. Blood (10 mL) samples for culture, haematological and biochemical testing, and stool culture samples were processed by the local hospital's accredited pathology laboratories as previously described.^19^ Quantitative blood culture (10 mL) was done at the time of diagnosis and processed according to the manufacturer's instructions (Alere, Waltham, MA, USA).

Secondary immunogenicity outcomes were post-vaccination anti-Vi IgG titres. Serum samples were collected at screening, pre-vaccination, and at the time of challenge and stored at −80°C. Anti-Vi IgG titres were measured using a commercial ELISA kit (VaccZyme, The Binding Site, Birmingham, UK) according to the manufacturer's guidelines. Anti-Vi IgG subclass titres (IgG1, IgG2, and IgG3) were assessed with Vi-coated plates and reagents supplied by The Binding Site using a protocol adapted from the commercial VaccZyme assay.

### Statistical analysis

Based on experience from previous typhoid challenge studies using the same challenge strain and conditions, the expected attack rate in the control group was 60–75%.^19^, ^20^ A sample size of 30 per group was calculated to provide 80% power to detect a protective effect of 60%, resulting in a reduction in attack rate from 65% to 26%, at 5% significance level (two-sided). To account for 15% dropout between randomisation and challenge, the sample size was increased to at least 35 per group. The study was not powered to directly compare the two Vi-vaccine groups with each other. The two Vi-vaccines were considered to be distinct treatments and therefore did not require adjustment for multiple comparisons.

Attack rates and 95% CIs were calculated for each vaccine group using the per-protocol population (participants who completed the 14 day challenge period) as the pre-specified primary analysis. The difference in attack rate between the Vi-TT or Vi-PS and control groups was analysed with Pearson's χ^2^ test. Vaccine efficacy was calculated as the percentage reduction in attack rate in the Vi-vaccinated group (Vi-TT or Vi-PS) compared with the control group. Time to diagnosis, time to development of fever, and time to bacteraemia were summarised using the Kaplan-Meier method, participants were censored on day 14 and group comparisons were done using a log-rank test. A post-hoc competing risk model for time to fever preceding a positive blood culture was fitted, in addition to a competing risks regression model with positive blood culture without fever treated as a competing risk. The model was fitted in Stata (version 14) using a maximum likelihood-based method based on Fine and Gray.^21^ Post-hoc analyses of vaccine efficacy using alternative diagnostic criteria were done in an attempt to better represent cases presenting in endemic settings (eg, fever with bacteraemia and fever thresholds) and were compared with Pearson's χ^2^ test or Fisher's exact test after initial data analysis review.

Vaccine immunogenicity was measured by ELISA (total anti-Vi IgG titre) and analysed using linear regression of log_10_ transformed antibody levels, adjusted for baseline titres. Total anti-Vi IgG titres less than the lower limit of detection of 7·4 ELISA units (EU)/mL (ie, undetectable titres) were assigned a value of 3·7 EU/mL before log transformation (half the lower limit of detection). Subclass titres were assigned 0·78 EU/mL if they were below the lower limit of detection of 1·56 EU/mL. Blood quantification CFUs less than 0·1 per mL were assigned a value of 0·05 per mL. Antibody titre and CFU comparisons between groups were analysed using Mann-Whitney U tests. A post-hoc analysis was done to assess the relation between anti-Vi IgG titre at the time of challenge (28 days post-vaccination) and typhoid diagnosis using logistic regression models with the independent variable of anti-Vi IgG titre at the time of challenge and the outcome of infection (based on the pre-specified composite definition of typhoid infection). The relationship was also explored using a model adjusting for vaccine group. Data were analysed using Stata 14 (StataCorp, TX, USA) and GraphPad Prism version 7.0.

An independent data and safety monitoring committee with access to unblinded data provided oversight of the safety and conduct of the trial. This trial is registered with ClinicalTrials.gov, number NCT02324751.

### Role of the funding source

The funders of the study had no role in study design, data collection, data analysis, data interpretation, or writing of the report. The first and last authors (CJ, AJP) had full access to all the data in the study and were responsible for the decision to submit for publication.

## Results

**Figure 1:**
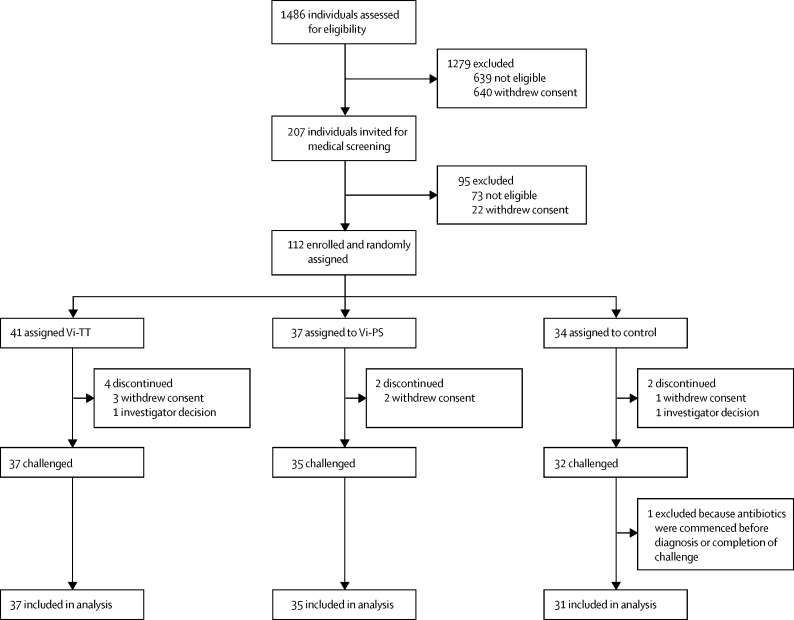
Trial profile Vi-TT=Vi-tetanus toxoid conjugate vaccine. Vi-PS=Vi-polysaccharide vaccine.

Between Aug 18, 2015, and Nov 4, 2016, 1486 participants were assessed for eligibility. Of the 207 volunteers who were screened, 112 were enrolled, randomly assigned, and vaccinated (73 failed to meet the eligibility criteria and 22 declined further study participation; figure 1). 41 participants were randomly assigned to the Vi-TT group, 37 to the Vi-PS group, and 34 to the control group. Eight participants withdrew before challenge (four in the Vi-TT group, two in the Vi-PS group, and two in the control group), six withdrew consent due to changes in personal circumstances affecting study visit availability, one participant was diagnosed with inflammatory bowel disease (symptoms preceded study enrolment), and one participant was excluded due to persistently increased alanine aminotransferase concentrations. Of the 104 participants who underwent challenge, 103 were included in the per-protocol analysis, one participant was excluded due to failure to complete the challenge period (antibiotics were given at the participant's request without a diagnosis of typhoid and before day 14 of challenge). Median time between vaccination and challenge was 28 days (range 25–40).

Baseline characteristics of participants were similar across vaccine groups, with 29–38% of participants having detectable anti-Vi IgG titres (table 1), which are likely to have arisen as a result of natural exposure to other bacteria expressing a Vi capsule (eg, *Citrobacter* spp) or through cross-reactive epitopes on other organisms.

**Table 1:** Baseline participant characteristics

		**Control group (n=34)**	**Vi-TT group (n=41)**	**Vi-PS group (n=37)**
Vaccinated		34	41	37
Sex				
	Women	10 (29%)	19 (46%)	13 (35%)
	Men	24 (71%)	22 (54%)	24 (65%)
Age (years)		31·3 (11·9)	31·2 (11·9)	33·8 (12·0)
Ethnic origin				
	Caucasian	33 (97%)	35 (85%)	35 (95%)
	Other	1 (3%)	6 (15%)	2 (5%)
Detectable baseline Vi-titre (>7·4 EU/mL)		13 (38%)	12 (29%)	12 (32%)

Data are n, n (%), or mean (SD). Ethnicity was self-reported. Vi-TT=Vi-tetanus toxoid conjugate vaccine. Vi-PS=Vi-polysaccharide vaccine.

**Figure 2:**
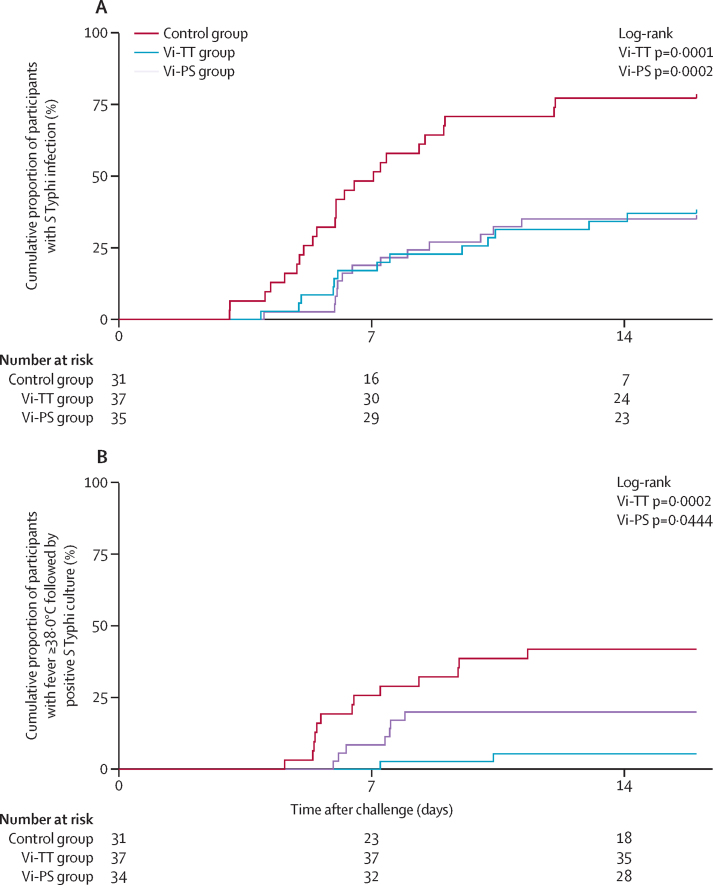
Proportion of participants diagnosed with typhoid infection (A) Cumulative proportion of participants with typhoid infection (meeting predefined microbiological, clinical criteria, or both) after *Salmonella* Typhi (*S Typhi*) challenge. (B) Cumulative proportion of participants with fever 38·0°C or higher preceding *S Typhi* bacteraemia (time to event measured from time of challenge agent ingestion to time of first positive blood culture sampling or time of first recorded fever ≥38·0°C). Vi-TT=Vi-tetanus toxoid conjugate vaccine. Vi-PS=Vi-polysaccharide vaccine.

Vaccination with Vi-TT significantly reduced the number of typhoid fever cases compared with control vaccination. Using the composite primary endpoint of bacteraemia or persistent fever, typhoid infection was diagnosed in 13 (35%) of 37 participants in the Vi-TT group and 24 (77%) of 31 in the control group (p=0·0005). The calculated vaccine efficacy of Vi-TT was 54·6% (95% CI 26·8–71·8; table 2; figure 2A). A significant reduction in typhoid fever cases was also observed in the Vi-PS group (attack rate 37% [13 of 35]; estimated vaccine efficacy 52·0% [95% CI 23·2–70·0]; p=0·0010).

**Table 2:** Primary and secondary outcomes

	**Control group (n=34)**	**Vi-TT group (n=41)**	**Vi-PS group (n=37)**
**Primary outcome**			
Completed challenged	31	37	35
Total diagnosed (composite definition, clinical or microbiological typhoid diagnosis)	24/31 (77%)	13/37 (35%)	13/35 (37%)
Relative risk (95% CI)	··	0·45 (0·28−0·73)	0·48 (0·30−0·77)
Vaccine efficacy (%, 95% CI)	··	54·6% (26·8–71·8)	52·0% (23·2–70·0)
p value	··	0·0005	0·0010
**Secondary outcomes**			
Time to diagnosis (days)	6·0 (5·1–7·8)	6·5 (6·1–8·6)	7·2 (5·9–10·2)
Microbiological diagnosis	16/31 (52%)	12/37 (32%)	9/35 (26%)
Time to microbiological diagnosis (days)	6·0 (4·6–8·0)	6·3 (6·0–8·3)	6·1 (5·1–10·2)
Clinical diagnosis	8/31 (26%)	1/37 (3%)	4/35 (11%)
Time to clinical diagnosis (days)	6·8 (5·4–7·8)	10·4	8·5 (6·5–10·0)
**Clinical outcomes**			
Fever ≥37·5°C (any duration)	20/31 (65%)	13/37 (35%)	18/35 (51%)
Fever ≥38·0°C (any duration)	17/31 (55%)	6/37 (16%)	11/35 (31%)
Fever ≥38·5°C (any duration)	14/31 (45%)	4/37 (11%)	9/35 (25%)
Time to first fever ≥38·0°C (any duration; days)	7·2 (5·4–8·5)	10·4 (10·2–15·5)	7·5 (6·2–8·7)
**Microbiological outcomes**			
*S* Typhi bacteraemia	24/24 (100%)	13/13 (100%)	11/13 (85%)
Time to first positive blood culture (days)	6·1 (5·0–7·6)	6·5 (6·1–8·6)	6·1 (5·0–10·2)
Participants with positive *S* Typhi stool culture	22/31 (71%)	22/37 (59%)	21/35 (60%)
Diagnosed participants with positive *S* Typhi stool culture	19/24 (79%)	12/13 (92%)	10/13 (77%)
Median quantitative blood culture (CFU/mL; range)	0·4 (0·05–22·7)	0·075 (0·05–1·2)	0·1 (0·05–5·6)

Data are n, n/N (%), or median (IQR) unless otherwise stated. Vi-TT=Vi-tetanus toxoid conjugate vaccine. Vi-PS=Vi-polysaccharide vaccine. *S* Typhi=*Salmonella* Typhi. CFU=colony forming units.

Clinical manifestations of typhoid fever seemed less severe among diagnosed participants following Vi-TT vaccination. Rates of fever of 38·0°C or higher were noted in six (16%) of 37 in the Vi-TT group, 17 (55%) of 31 in the control group, and 11 (31%) of 35 in the Vi-PS group (table 2), and peak median C-reactive protein concentrations in diagnosed participants were 31·2 mmol/L (IQR 11·0–58·8) in the Vi-TT group, 52·2 mmol/L (23·2–74·5) in the Vi-PS group, and 45·6 mmol/L (27·2–91·7) in the control group (appendix p 6). In addition, fewer Vi-TT diagnosed participants reported severe symptoms when comparing solicited typhoid symptoms following challenge (three [23%] of 13 in the Vi-TT group, six [46%] of 13 in the Vi-PS group, and 14 [58%] of 24 in the control group; appendix pp 4–5).

Post-hoc analyses of alternative diagnostic criteria, such as fever of 38·0°C or higher preceding *S* Typhi bacteraemia, showed significant differences between the Vi-vaccinated and control groups (figure 2B) and resulted in vaccine efficacy estimates of 87·1% (95% CI 47·2 to 96·9) for Vi-TT and 52·3% (−4·2 to 78·2) for Vi-PS (appendix p 1). The results remained unchanged after fitting a post-hoc competing risk model for time to fever preceding a positive blood culture, and a competing risks regression model with positive blood culture without fever treated as a competing risk.

All diagnosed participants, except for two participants in the Vi-PS group, had *S* Typhi detected from blood culture during the challenge period. Median time to first positive blood culture was 6·1 days (IQR 5·0–7·6) for the control group, 6·5 days (6·1–8·6) for the Vi-TT group, and 6·1 days (5·0–10·2) for the Vi-PS group (table 2). Median number of *S* Typhi CFUs detected by quantitative blood culture, collected before antibiotic commencement in typhoid diagnosed participants, was reduced in both Vi-vaccine groups, and significantly reduced in participants in the Vi-TT group (median 0·075 CFU/mL [range 0·05–1·2]) compared with control participants (0·4 CFU/mL [0·05–22·7]; p=0·0476). *S* Typhi was detected from stool cultures in diagnosed and undiagnosed participants irrespective of vaccine assignment (table 2).

**Figure 3:**
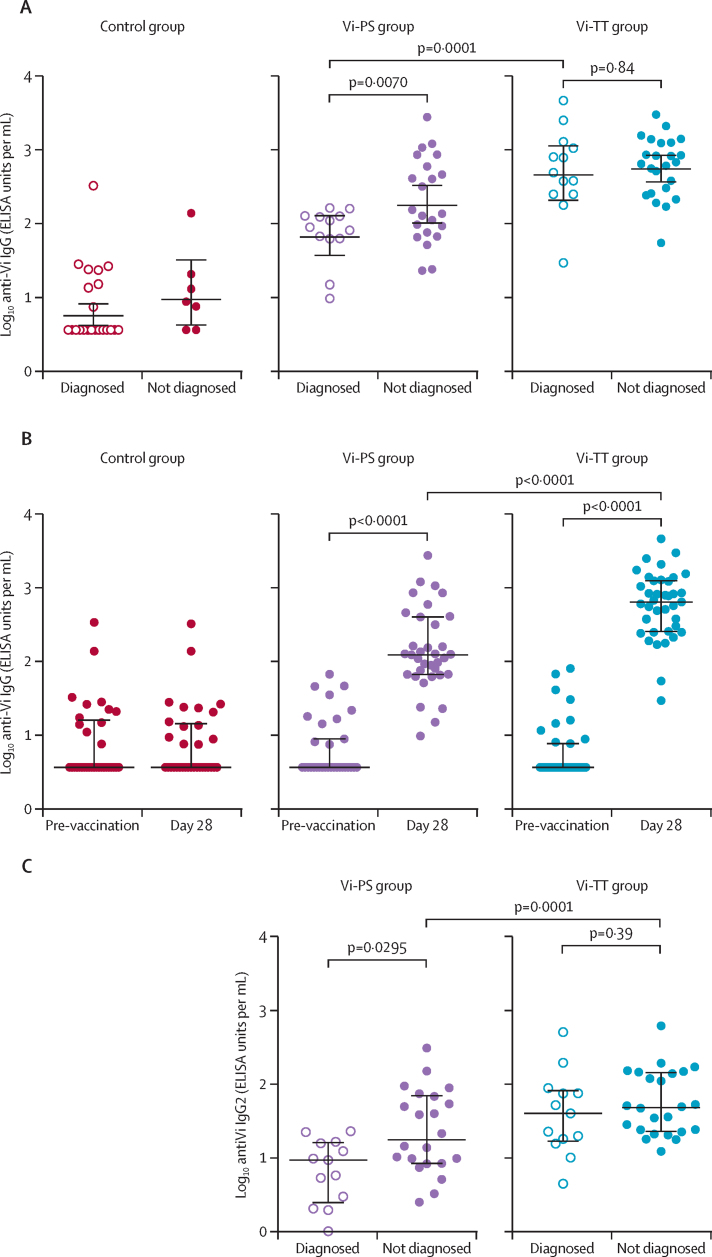
Anti-Vi IgG titres measured following vaccination (A) Anti-Vi IgG titres measured at the time of challenge, comparison between typhoid diagnosed (open symbols) and undiagnosed (closed symbols). (B) Anti-Vi IgG titres measured at baseline (pre-vaccination) and at the time of challenge (median 28 days post-vaccination). (C) Anti-Vi IgG2 titres measured at the time of challenge in Vi-TT and Vi-PS diagnosed (open symbols) and undiagnosed (closed symbols). Vi-TT=Vi-tetanus toxoid conjugate vaccine. Vi-PS=Vi-polysaccharide vaccine.

Seroconversion (≥four-fold rise in antibody titre 28 days after vaccination) was 100% in the Vi-TT group and 88·6% in the Vi-PS group. Vi-TT vaccination induced significantly higher anti-Vi IgG titres than Vi-PS, geometric mean titre (GMT) adjusted for baseline of 562·9 EU/mL (95% CI 396·9–798·4) versus 140·5 EU/mL (91·0–216·9; p<0·0001; figure 3B; appendix p 2). Furthermore, significantly higher titres of all three measured IgG subclasses 1, 2, and 3 were induced by Vi-TT than by Vi-PS (appendix p 2). Despite having higher anti-Vi IgG titres overall, no significant differences in titres were detected between Vi-TT diagnosed and undiagnosed participants for total anti-Vi IgG or IgG subclasses (appendix p 2; figure 3A). By contrast, anti-Vi IgG titres were significantly higher in undiagnosed Vi-PS vaccinees than in diagnosed participants (mean GMT 207·5 EU/mL [95% CI 116–372] *vs* 72·8 EU/mL [43–122]; p=0·0070), in particular, anti-Vi IgG2 subclass antibodies were significantly higher in Vi-PS protected participants than in diagnosed participants (p=0·0295; appendix p 2; figure 3C).

From adjusted logistic regression, the odds of typhoid diagnosis were significantly lower in those participants with higher anti-Vi IgG titres than in participants with lower anti-Vi IgG titres (odds ratio [OR] 0·37 [95% CI 0·15–0·88]; p<0·0001). The results remained similar without adjustment for vaccine group (OR 0·35 [95% CI 0·21–0·59]; p=0·0001; appendix p 7).

Following vaccination, five participants reported low grade fever less than 38·0°C: two (6%) in the control group, two (5%) in the Vi-PS group, and one (2%) in the Vi-TT group. Of the solicited post-vaccination symptoms, injection site pain was more commonly reported in the participants in the Vi-PS group (33 [89%]) and Vi-TT group (25 [61%]) than in the control group (13 [38%]; p<0·0001 and p=0·0499, respectively). Four serious adverse events were reported during the conduct of the study (one [2%] in the Vi-TT group and three [8%] in the Vi-PS group), none of which were related to vaccine administration (appendix p 3).

## Discussion

This is the first clinical trial to show that immunisation with a Vi-TT vaccine is safe, well tolerated, and halves the total number of typhoid infection cases, in the context of a controlled human infection model of typhoid fever. While the calculated vaccine efficacy of 54·6% for Vi-TT is encouraging, it is likely that the protective effect of Vi-TT in endemic settings is higher. The protective effect of Vi-PS identified from our study was similar to Vi-TT at 52%; however, this is notably less than that observed in field trials of Vi-PS, where efficacy is estimated at 69% within the first year following vaccination.^11^ This discrepancy in vaccine efficacy might be partly due to differences in participant populations, with healthy typhoid-naive adults enrolled in the challenge study versus pre-exposed individuals, including children living in endemic settings.

Historical typhoid vaccine challenge studies done by Woodward and colleagues^22^ 60 years ago found that larger inocula of *S* Typhi (10^6^ or 10^7^ CFUs) produced higher attack rates (65%), but overwhelmed protection afforded by known efficacious vaccines (eg, the whole-cell inactivated parenteral vaccine). Because the infecting dose of *S* Typhi in endemic settings is unknown, the inoculum used in this study, 10^4^ CFUs, combined with the co-administration of sodium bicarbonate buffer to increase susceptibility to infection through gastric acid suppression, probably represents a high infecting inoculum, as suggested by the high attack rate (77%) in the control group. In view of this high attack rate, it is plausible that vaccine-induced immunity in some Vi-vaccinees was overwhelmed by the large bacterial inoculum resulting in infection, despite the presence of Vi antibody.

The use of a composite definition of typhoid fever, to capture microbiological cases, and clinical presentations was necessary to ensure that all potential cases of infection were identified and treated promptly with antibiotics to safeguard participants. This all-encompassing definition of typhoid could have included participants with self-resolving asymptomatic bacteraemias who would otherwise have remained undetected in field efficacy studies.^23^ As such, inclusion of these participants as diagnosed cases might have reduced vaccine efficacy estimates. The diagnostic criteria used in our model were not designed to mirror field trial definitions of typhoid fever. However, if an approximate field definition of typhoid fever were applied, such as fever 38·0°C or higher followed by bacteraemia, the estimated efficacy of Vi-TT would be 87·1%. Although these analyses were done post hoc, this definition of typhoid fever has also been used to estimate vaccine efficacy in a previous typhoid challenge study using Ty21a.^20^ The estimated efficacy was 80% (95% CI 16–95), similar to the original challenge studies using Ty21a, and field studies, which might suggest that efficacy estimates using this endpoint more accurately reflect the efficacy of typhoid vaccines in endemic settings.^12^, ^22^, ^24^ Furthermore, the estimate of 87·1% is similar to that found with the Vi-rEPA conjugate vaccine, which provided 89% protection against blood-culture-positive typhoid fever cases in Vietnamese children aged between 2 and 5 years receiving two vaccine doses.^11^, ^15^

A further observation, which was also noted in the Vi-rEPA efficacy study,^15^ was that clinical manifestations of typhoid disease seemed less severe among those who developed typhoid fever despite Vi-TT vaccination. In addition, the lower blood bacterial load in these individuals at the time of diagnosis, when compared with control participants, might suggest that after ingesting a high bacterial inoculum, the presence of Vi-antibody reduces the number of invading organisms or bacterial replication during the incubation period, resulting in an attenuated disease profile. This finding has also been observed in a previous typhoid vaccine challenge study using a live attenuated oral vaccine, M01ZH09.^20^ A reduction in the severity of typhoid symptoms could lead to fewer undifferentiated fever presentations to pharmacies in high-burden settings, which would reduce inappropriate antibiotic use and assist with the management of the growing problem of *S* Typhi AMR.

Protection against *S* Typhi following Vi-vaccination is primarily antibody mediated.^25^ In the Vi-PS group, those participants diagnosed with typhoid had lower total anti-Vi IgG titres, and in particular lower IgG2 titres, the main subclass known to target polysaccharide antigens after immunisation with plain polysaccharides,^26^ suggesting that antibody quantity and perhaps the type of IgG subclass induced by Vi-PS vaccination play an important part in protecting against disease. Interestingly, no significant difference in titres between diagnosed and undiagnosed participants was identified in the Vi-TT group, suggesting that antibody functionality is equally important for protection as absolute antibody quantity. Future work assessing functional Vi-antibody (such as those mediating bactericidal, opsonophagocytic, or antibody-dependent cellular-cytotoxicity activity) is required to interrogate differences in antibody quality in protected individuals versus susceptible individuals.

Logistic regression modelling of the relation between anti-Vi IgG titre and the risk of developing typhoid disease suggests that higher anti-Vi IgG titres are associated with a lower risk of disease. Despite this, an absolute threshold of protection, above which individuals are 100% protected from disease, could not be identified from these data. This might be a phenomenon unique to the typhoid challenge model, a by-product of using a high *S* Typhi inoculum that might overwhelm vaccine-induced protection. However, it could also be related to the pathogenesis of typhoid disease and mechanisms of host evasion. *S* Typhi is primarily an intracellular bacterium that highly regulates Vi-capsule expression.^27^, ^28^ Differences in innate or mucosal host responses could account for variations in susceptibility to infection, despite the presence of large quantities of anti-Vi IgG antibody, and further investigation of these responses is warranted. In addition, the mechanism of action of Vi antibody following vaccination might be related to lowering the infectious dose or degree of bacterial replication, rather than sterilising immunity at the mucosal surface, as indicated by experience with other polysaccharide-conjugate vaccines. By showing a relation between anti-Vi IgG titre and the risk of typhoid disease, we have shown that post-vaccination anti-Vi IgG titres will be a key component for assessing vaccine immunogenicity, particularly as more Vi-conjugate vaccines enter later stages of clinical testing. Evaluation from field trials in relevant endemic settings, such as those led by the Typhoid Vaccine Acceleration Consortium (TyVAC), will assist with validating these findings.

Logistical, ethical, and safety considerations in the study design limit the extent to which our findings can be extrapolated to typhoid endemic countries. Volunteers cannot be left untreated indefinitely, which potentially leads to underestimation of the attack rate. Furthermore, early treatment could prevent identification of self-limited infection and lead to an over-estimation of the clinical attack rate. Notably, the study population (healthy adult volunteers with no known previous exposure to typhoid infection) is not wholly representative of the populations where typhoid fever is endemic and Vi-TT might eventually be deployed. Nevertheless, immunogenicity in children immunised from 2 months of age,^12^ antibody persistence up to 8 years following vaccination,^29^ detection of higher avidity Vi-antibody,^14^ and boosting of antibody titres with re-vaccination 2 years after priming^14^ have all been shown using different TCVs in high-burden settings and contrast with findings for Vi-PS. Limitations of the currently available typhoid vaccines, in particular the absence of a licensed vaccine for children younger than 2 years of age, have contributed to the poor adoption of typhoid immunisation programmes in endemic settings despite a WHO recommendation in 2008 supporting their use. Further efficacy data from phase 3 studies in this young age group, in addition to cost-effectiveness studies and the assessment of direct versus indirect protection, will support the introduction of TCVs. For now, the efficacy results from our challenge study can be combined with the growing body of immunological evidence supporting the use of TCVs, and can be considered by both key decision makers and adopting countries when evaluating the future use of TCVs in typhoid endemic regions.

Typhoid vaccine candidates have been evaluated using experimental human challenge studies since the 1960s^22^ and have successfully supported the development of one of the currently licensed typhoid vaccines, Ty21a.^24^ 60 years on, we have used a controlled human infection model of typhoid fever to show that a Vi-tetanus toxoid conjugate vaccine is safe, immunogenic, and prevents at least 55% of typhoid fever cases, though calculations of vaccine efficacy within the confines of a stringent challenge model are likely to be conservative estimates. This human challenge study provides further evidence to support the deployment of Vi-conjugate vaccines as a control measure to reduce the burden of typhoid fever because those individuals living in endemic regions should not be made to wait another 60 years.
