# Supplementary material for: Efficacy and immunogenicity of a Vi-tetanus toxoid conjugate vaccine in the prevention of typhoid fever using a controlled human infection model of Salmonella Typhi: a randomised controlled, phase 2b trial
Source: Lancet. 2017 Dec 2;390(10111):2472–80. doi: 10.1016/S0140-6736(17)32149-9 (PMC5720597; doi:10.1016/S0140-6736(17)32149-9)
Supplement: Supplementary appendix [file mmc1.pdf]

THE LANCET

Supplementary appendix

This appendix formed part of the original submission and has been peer reviewed. We post it as supplied by the authors.

Supplement to: Jin C, Gibani MM, Moore M. Efficacy and immunogenicity of a Vi-tetanus toxoid conjugate vaccine in the prevention of typhoid fever using a controlled human infection model of *Salmonella* Typhi: a randomised controlled, phase 2b trial. *Lancet* 2017; published online Sept 28. http://dx.doi.org/10.1016/S0140-6736(17)32149-9.

SUPPLEMENTARY DATA

Table S1. – Alternative Clinical Diagnostic Criteria (Post-hoc analyses)

	**Control** (n=31)	**Vi-TT** (n=37)	**Vi-PS** (n=35)
**Fever ≥37·5˚C (any duration) + bacteraemia***	18/31 (58%)	7/37 (19%)	9/35 (26%)
Relative Risk		0·33 (0·16, 0·68)	0·44 (0·23, 0·84)
Vaccine Efficacy %		67·4% (32·3%, 84·3%)	55·7% (16·2%, 76·6%)
P-value		0·0009	0·0076
**Fever ≥38·0˚C (any duration) + bacteraemia***	16/31 (52%)	4/37 (11 %)	8/35 (23%)
Relative Risk		0·21 (0·08, 0·56)	0·44 (0·22, 0·89)
Vaccine Efficacy %		79·1% (43·8%, 92·2%)	55·7% (11·0%, 78·0%)
P-value		0·0003	0·0154
**Fever ≥38·0˚C (any duration) with *subsequent* bacteraemia**	13/31 (42%)	2/37 (5%)	7/35 (20%)
Relative Risk		0·13 (0·03, 0·53)	0·48 (0·22, 1·04)
Vaccine Efficacy %		87·1% (47·2%, 96·9%)	52·3% (-4·2%, 78·2%)
P-value		0·0004	0·05

*Fever (of a particular threshold) + bacteraemia = any positive blood culture and any fever occurring in the same individual during the 14 day challenge period (i.e. no temporal relationship required between two events).

Data are n/N (%), 95% Confidence Intervals are represented for relative risk and vaccine efficacy calculations.

Table S2. – Anti-Vi specific antibody responses

	**Control**		**Vi-TT**	**P value** (Control vs Vi-TT)	**Vi-PS**	**P value** (Control vs Vi-PS)	**P value** (Vi-TT vs Vi-PS)
**Pre-vaccination GMT (95% CI)**	7·7 (5·0, 12·0)		5·6 (4·2, 7·5)	0·27	6·3 (4·3, 8·7)	0·64	0·50
**Day 28 GMT (95% CI)**							
**Total Anti-Vi IgG**	8·0 (5·2, 12·2)		562·9 (396·9, 798·8)	<0·0001	140·5 (91·0, 216·9)	<0·0001	<0·0001
		**P Value** (Control Diagnosed vs Undiagnosed)		**P Value** (Vi-TT Diagnosed vs Undiagnosed)		**P Value** (Vi-PS Diagnosed vs Undiagnosed)	**P value** (Vi-TT vs Vi-PS)
Diagnosed	7·1 (4·4, 11·4)	0·19	522 (241, 1129)	0·84	73 (43, 122)	0·0070	
Undiagnosed	12·0 (3·8, 37·9)		586 (396, 868)		207 (116, 372)		
**Anti-Vi IgG1**			85·6 (61·5, 119·2)		14·0 (9·2, 21·3)		<0·0001
Diagnosed			85·9 (40·2, 183·8)	0·75	8·9 (5·9, 13·3)	0·10	
Undiagnosed			85·4 (59·7, 122·1)		18·4 (9·9, 34·0)		
**Anti-Vi IgG2**			49·0 (33·8, 71·1)		15·1 (9·8, 23·2)		0·0001
Diagnosed			39·2 (17·8, 86·2)	0·39	7·8 (4·9, 12·3)	0·0295	
Undiagnosed			55·3 (36·1, 84·8)		22·3 (12·4, 40·1)		
**Anti-Vi IgG3**			116·6 (84·5, 161·0)		27·6 (18·9, 40·4)		<0·0001
Diagnosed			96·8 (47·0, 199·4)	0·55	20·2 (15·4, 26·5)	0·17	
Undiagnosed			129·0 (90·1, 183·1)		33·2 (18·4, 59·9)		

Table S3. Safety - Vaccine Reactogenicity and Serious Adverse Event Reporting
Solicited symptoms (of any severity) for 7 days following vaccination.

	**Control (n=34)**	**Vi-TT (n=41)**	**Vi-PS (n=37)**
Fever ≥37·5°C and <38·0°C	2 (5·9%)	1 (2·4%)	2 (5·4%)
Headache	9 (26·5%)	14 (34·1%)	12 (32·4%)
Malaise	7 (20·6%)	9 (22%)	4 (10·8%)
Anorexia	2 (5·9%)	3 (7·3%)	3 (8·1%)
Myalgia	8 (23·5%)	13 (31·7%)	8 (21·6%)
Arthralgia	5 (14·7%)	3 (7·3%)	4 (10·8%)
Injection Site Pain	13 (38·2%)	25 (61%)*	33 (89·2%)**
Injection Site Erythema	0	1 (2·4%)	1 (2·7%)
Injection Site Induration	0	0	0
Injection Site Swelling	0	1 (2·4%)	0
Serious Adverse Events†	0	1	3

Data are n (%),*P=0·0499, **P<0·0001

†Four Serious Adverse Events were reported to the Data Safety and Monitoring Committee during the conduct of the study, however none were related to vaccination and blinding was maintained. One Vi-TT participant was diagnosed with inflammatory bowel disease and withdrawn from the study (symptoms preceded study enrolment), two Vi-PS participants were hospitalised (urinary retention and semi-elective tonsillectomy) and one Vi-PS participant was diagnosed with reactive arthritis possibly related to *S.* Typhi challenge or antibiotic treatment.

Table S4. Post-challenge symptom severity scores according to vaccine group

	Control	Vi-TT	Vi-PS
**Headache (n)**			
Mild	4	3	2
Moderate	7	5	6
Severe	12	3	4
**Malaise (n)**			
Mild	4	1	2
Moderate	11	6	3
Severe	8	2	6
**Anorexia (n)**			
Mild	10	6	2
Moderate	6	3	6
Severe	4	1	0
**Abdominal Pain (n)**			
Mild	9	8	5
Moderate	6	3	2
Severe	4	0	2
**Nausea/Vomiting (n)**			
Mild	8	4	4
Moderate	3	0	1
Severe	2	1	2
**Myalgia (n)**			
Mild	5	2	4
Moderate	7	5	1
Severe	3	1	2
**Arthralgia (n)**			
Mild	9	1	2
Moderate	3	5	3
Severe	4	0	0
**Constipation (n)**			
Mild	7	1	7
Moderate	2	3	0
Severe	2	3	0
**Diarrhoea (n)**			
Mild	5	2	1
Moderate	0	1	0
Severe	0	0	0
**Cough (n)**			
Mild	7	7	2
Moderate	1	0	2
Severe	1	0	0

Total (n, %) – Any symptom	Control	Vi-TT	Vi-PS
No Symptoms	0 (0%)	2 (15·4%)	0 (0%)
Mild	3 (12·5%)	1 (7·7%)	2 (15·4%)
Moderate	7 (29·2%)	7 (53·8%)	5 (38·5%)
Severe	14 (58·3%)	3 (23·1%)	6 (46·2%)

Figure S1. Maximum severity of typhoid symptoms for diagnosed participants according to vaccine group.

Solicited symptoms were collected for 21 days following challenge. Severe = significant interference with daily activities requiring codeine analgesia, Moderate = some interference with daily activities not requiring codeine analgesia, Mild = minor interference with daily activities

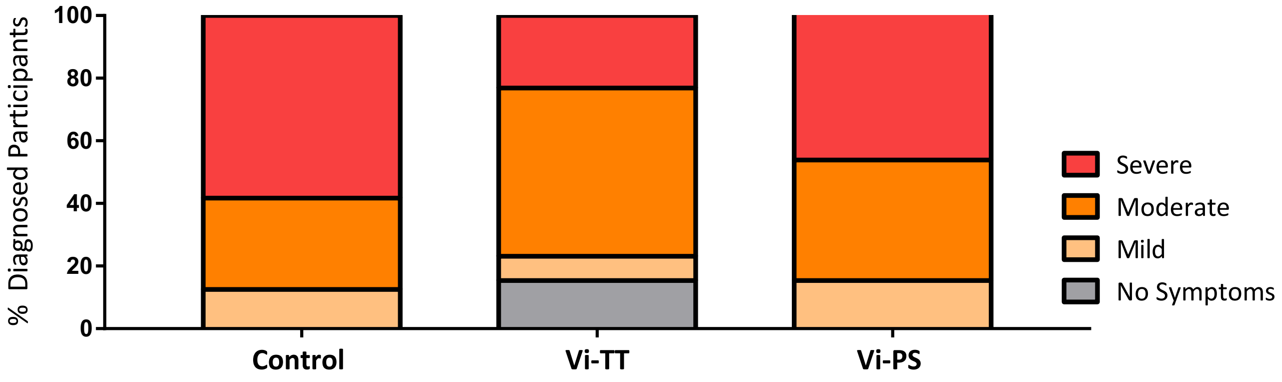

Figure S2. – Median C-reactive protein (CRP) level in typhoid diagnosed participants according to vaccine group

Median CRP (solid line), IQR (dashed lines), time point 0 represents time of antibiotic commencement.

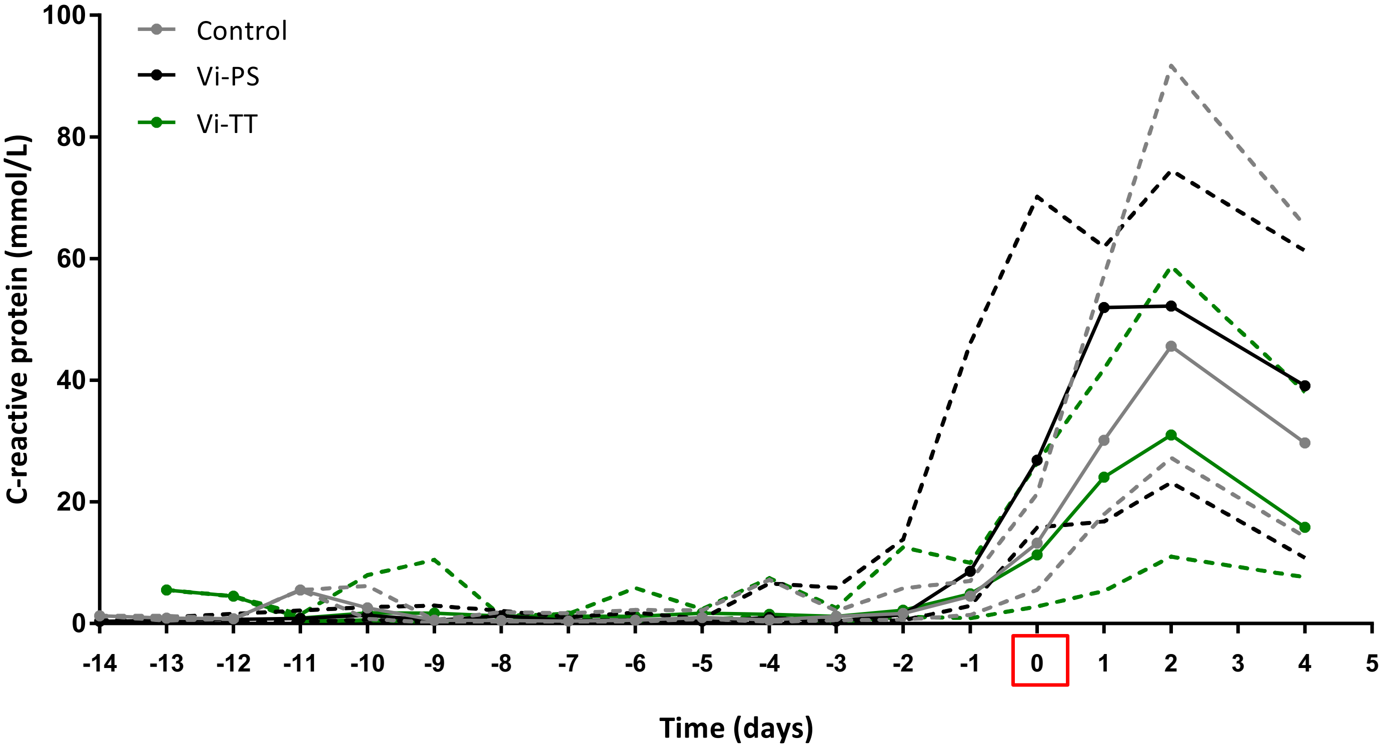

Figure S3. Logistic regression modelling of relationship between anti-Vi IgG titre and probability of typhoid diagnosis

(D0 = day of challenge/day of typhoid exposure).

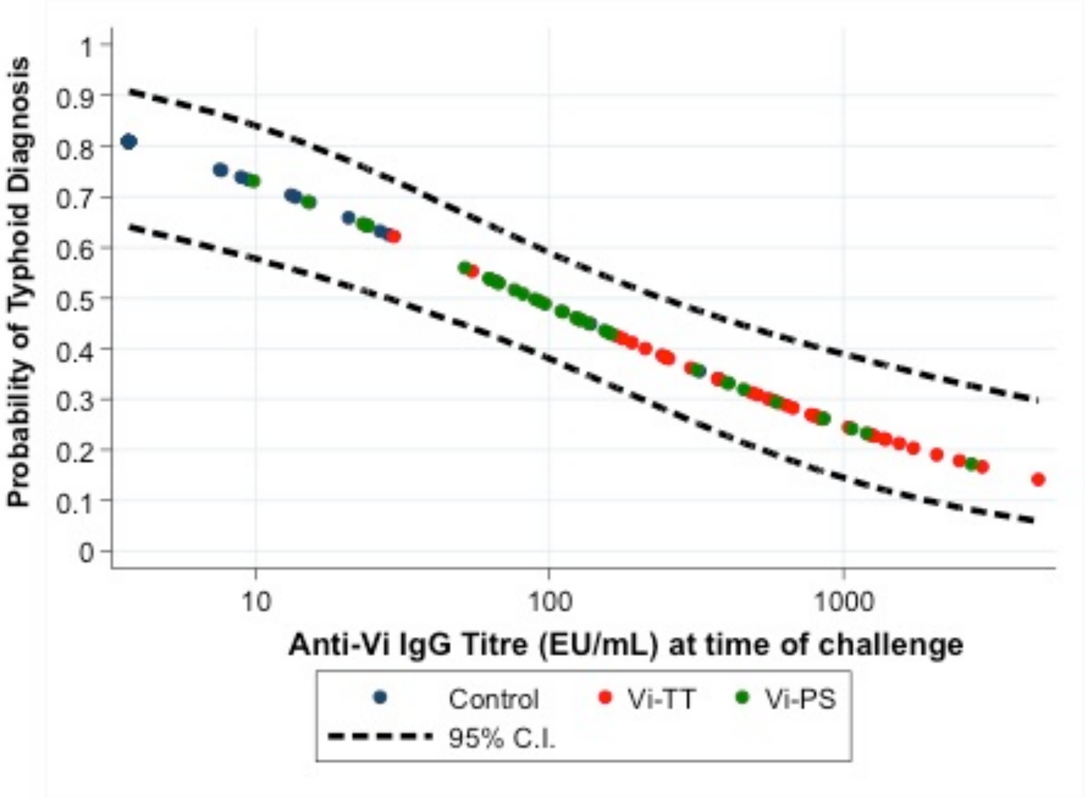

Supplementary Methods – Inclusion and Exclusion Criteria

Inclusion Criteria

Participants must satisfy all of the following criteria to be considered eligible for the study:

- Agree to give informed consent for participation in the study.
- Aged between 18 and 60 years inclusive at time of vaccination.
- In good health as determined by medical history, physical examination and clinical judgment of the study team.
- Agree (in the study team's opinion) to comply with all study requirements, including capacity to adhere to good personal hygiene and infection control precautions.
- Agree to allow his or her General Practitioner (and/or Consultant if appropriate), to be notified of participation in the study.
- Agree to allow study staff to contact his or her GP to access the participant's vaccination records.
- Agree to allow Public Health England to be informed of their participation in the study.
- Agree to give his or her close contacts written information informing them of the participant's involvement in the study and offer them voluntary screening for *S.* Typhi carriage.
- Agree to have 24-hour contact with study staff during the four weeks post challenge and are able to ensure that they are contactable by mobile phone for the duration of the challenge period until antibiotic completion.
- Agree to allow the study team to hold the name and 24-hour contact number of a close friend, relative or housemate who will be kept informed of the study participant's whereabouts for the duration of the challenge period (from the time of challenge until completion of antibiotic course). This person will be contacted if study staff are unable to contact the participant.
- Have internet access to allow completion of the e-diary and real-time safety monitoring.
- Agree to avoid antipyretic/anti-inflammatory treatment from the time of challenge (Day 0) until advised by a study doctor or until 14 days after challenge.
- Agree to refrain from donating blood for the duration of the study
- Agree to provide their National Insurance/Passport number for the purposes of TOPS registration and for payment of reimbursement expenses.

Exclusion Criteria

The participant will not be enrolled if any of the following apply:

- History of significant organ/system disease that could interfere with trial conduct or completion. Including, for example, but not restricted to:
    - Cardiovascular disease
    - Respiratory disease
    - Haematological disease
    - Endocrine disorders

    - Renal or bladder disease, including history of renal calculi
    - Biliary tract disease, including biliary colic, asymptomatic gallstones or previous cholecystectomy
    - Gastro-intestinal disease including requirement for antacids, H_2-receptor antagonists, proton pump inhibitors or laxatives
    - Neurological disease
    - Metabolic disease
    - Autoimmune disease
    - Psychiatric illness requiring hospitalisation or known or suspected drug and/or alcohol misuse (alcohol misuse defined as an intake exceeding 42 units per week)
    - Infectious disease
- Have any known or suspected impairment of immune function, alteration of immune function, or prior immune exposure that may alter immune function to typhoid resulting from, for example:
    - Congenital or acquired immunodeficiency, including IgA deficiency
    - Human Immunodeficiency Virus infection or symptoms/signs suggestive of an HIV-associated condition
    - Receipt of immunosuppressive therapy such as anti-cancer chemotherapy or radiation therapy within the preceding 12 months or long-term systemic corticosteroid therapy
    - Receipt of immunoglobulin or any blood product transfusion within 3 months of study start.
    - History of cancer (except squamous cell or basal cell carcinoma of the skin and cervical carcinoma in situ).
- Moderate or severe depression or anxiety as classified by the Hospital Anxiety and Depression Score at screening or challenge that is deemed clinically significant by the study doctors.
- Weight less than 50kg.
- Presence of implants or prosthesis.
- Anyone taking long-term medication (e.g. analgesia, anti-inflammatories or antibiotics) that may affect symptom reporting or interpretation of the study results.
- Contraindication to ciprofloxacin or macrolide antibiotics.
- Female participants who are pregnant*,* lactating or who are unwilling to ensure that they or their partner use effective contraception 30 days prior to vaccination and continue to do so until two negative stool samples, a minimum of 3 weeks after completion of antibiotic treatment, have been obtained.
- Full-time, part-time or voluntary occupations involving:
    - Clinical or social work with direct contact with young children (defined as those attending pre-school groups or nursery or aged under 2 years), or
    - Clinical or social work with direct contact with highly susceptible patients or persons in whom typhoid infection would have particularly serious consequences (unless willing to avoid work until demonstrated not to be infected with *S*. Typhi in

accordance with guidance from Public Health England and willing to allow study staff to inform their employer).

- Full time, part time or voluntary occupations involving:
    - Commercial food handling (involving preparing or serving unwrapped foods not subjected to further heating)
- Close household contact with:
    - Young children (defined as those attending pre-school groups, nursery or those aged less than 2 years)
    - Individual(s) who is (are) immunocompromised.
- Scheduled elective surgery or other procedures requiring general anaesthesia during the study period.
- Participants who have participated in another research study involving an investigational product that might affect risk of typhoid infection or compromise the integrity of the study within the 30 days prior to enrolment (e.g. significant volumes of blood already taken in previous study).
- Detection of any abnormal results from screening investigations (at the clinical discretion of the study team).
- Inability to comply with any of the study requirements (at the discretion of the study staff and the participant's General Practitioner).
- Any other social, psychological or health issues which, in the opinion of the study staff, may
    - Put the participant or their contacts at risk because of participation in the study,
    - Adversely affect the interpretation of the primary endpoint data,
    - Impair the participant's ability to participate in the study.
- Having previously received any typhoid vaccine
- Having been resident in an enteric fever endemic country for 6 months or more.
- Have previously been diagnosed with laboratory-confirmed typhoid or paratyphoid infection or been given a diagnosis compatible with enteric fever.
- Have participated in previous typhoid or paratyphoid challenge studies (with ingestion of challenge agent).
- Have received vaccination with a vaccine containing tetanus toxoid within the past 12 months.
- Have any history of allergy to vaccine components (including tetanus toxoid and diphtheria CRM protein).
- Have a prolonged corrected QT interval (>450 milliseconds) on ECG screening.
